# The Progress of Stem Cell Technology for Skeletal Regeneration

**DOI:** 10.3390/ijms22031404

**Published:** 2021-01-30

**Authors:** Shoichiro Tani, Hiroyuki Okada, Ung-il Chung, Shinsuke Ohba, Hironori Hojo

**Affiliations:** 1 Sensory & Motor System Medicine, Graduate School of Medicine, The University of Tokyo, 7-3-1 Hongo, Bunkyo-ku, Tokyo 113-8655, Japan; stani-tky@umin.ac.jp (S.T.); hokada-tky@umin.ac.jp (H.O.); 2 Center for Disease Biology and Integrative Medicine, Graduate School of Medicine, The University of Tokyo, Tokyo 113-0033, Japan; helixcm1@g.ecc.u-tokyo.ac.jp; 3 Department of Bioengineering, Graduate School of Engineering, The University of Tokyo, Tokyo 113-8656, Japan; 4 Department of Cell Biology, Institute of Biomedical Sciences, Nagasaki University, Nagasaki 852-8588, Japan; s-ohba@nagasaki-u.ac.jp

**Keywords:** stem cell, mesoderm, neural crest, skeletal regeneration

## Abstract

Skeletal disorders, such as osteoarthritis and bone fractures, are among the major conditions that can compromise the quality of daily life of elderly individuals. To treat them, regenerative therapies using skeletal cells have been an attractive choice for patients with unmet clinical needs. Currently, there are two major strategies to prepare the cell sources. The first is to use induced pluripotent stem cells (iPSCs) or embryonic stem cells (ESCs), which can recapitulate the skeletal developmental process and differentiate into various skeletal cells. Skeletal tissues are derived from three distinct origins: the neural crest, paraxial mesoderm, and lateral plate mesoderm. Thus, various protocols have been proposed to recapitulate the sequential process of skeletal development. The second strategy is to extract stem cells from skeletal tissues. In addition to mesenchymal stem/stromal cells (MSCs), multiple cell types have been identified as alternative cell sources. These cells have distinct multipotent properties allowing them to differentiate into skeletal cells and various potential applications for skeletal regeneration. In this review, we summarize state-of-the-art research in stem cell differentiation based on the understanding of embryogenic skeletal development and stem cells existing in skeletal tissues. We then discuss the potential applications of these cell types for regenerative medicine.

## 1. Introduction

Skeletal disorders, such as osteoarthritis and bone fractures, cause critical deformation and dysfunctions in skeletal tissues, resulting in a compromised quality of life, especially in elderly individuals [1,2]. Bone tissue has limited healing potential, but it can regenerate itself if the quantity and quality of the injury are not too severe. In the case of severe fractures or massive bone loss, the injuries cannot be repaired sufficiently and lead to non-union or pseudoarticulation [2]. On the other hand, articular cartilage has little or almost no potential for healing. The present therapeutic options for disorders involving the articular cartilage are predominantly palliative, and include analgesics and anti-inflammatory medication; no options can heal them effectively [3]. Thus, regenerative therapies using skeletal cells or their progenitors are recognized as important therapeutic alternatives [4].

**Figure 1:**
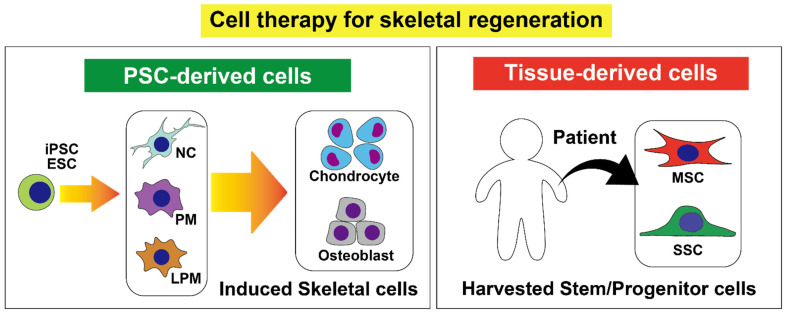
Summary of cell therapy in skeletal regeneration. PSC, pluripotent stem cell; iPSC, induced pluripotent stem cell; ESC, embryonic stem cell; NC, neural crest; PM, paraxial mesoderm; LPM, lateral plate mesoderm; MSC, mesenchymal stem/stromal cell; SSC, skeletal stem cell.

Currently, there are two major strategies for such cell therapies (Figure 1). The first is to use induced pluripotent stem cells (iPSCs) or embryonic stem cells (ESCs), which can recapitulate the skeletal developmental process and differentiate into various skeletal cells. Because skeletal tissues are derived from multiple origins, multiple protocols have been established based on the understanding of the skeletal development [5,6,7]. The second strategy is to use stem cells purified from adult skeletal tissues. The extracted cells are usually expanded and either locally implanted or intravascularly infused [8,9]. Mesenchymal stem/stromal cells (MSCs) are a conventional cell type that has been studied and applied to clinical settings [10]. In addition, multiple cell types including skeletal stem cells (SSCs) and CXCL12-abundant reticular (CAR) cells have been identified as cell populations with unique properties and potentials. These cells have distinct multipotent properties which allow them to differentiate into several skeletal cell types [11,12]. In this review, we first summarize the current understanding of skeletal development in embryos and the induction protocols for recapitulating the developmental process with stem cells. We then introduce MSCs and the other stem/progenitor cells in skeletal tissues. Lastly, we discuss the current limitations and potential applications of these cell types for regenerative medicine.

## 2. Skeletal Development in Embryos

Skeletal tissues originate from three distinct embryonic components according to the location: the neural crest, paraxial mesoderm, and lateral plate mesoderm [13]. The axial skeleton is derived from the paraxial mesoderm, whereas the appendicular skeleton originates from the lateral plate mesoderm. Most of the craniofacial skeleton is derived from the neural crest [13]. There are two modes of bone formation: intramembranous ossification and endochondral ossification. In intramembranous ossification, mesenchymal cells directly differentiate into osteoblasts that produce the bone. In endochondral ossification, bone formation occurs sequentially after cartilage formation [13]. The neural crest generates most of the craniofacial skeleton via intramembranous ossification, with a few exceptions, including the skull base. However, the paraxial and lateral plate mesoderm form most bones via endochondral ossification [13]. Based on the origins or mode of bone formation, different induction protocols have been established to generate skeletal cells from human pluripotent stem cells (hPSCs) [6,14,15]. Thus, we succinctly overview the current understanding of skeletal development and introduce each induction method.

### 2.1. Paraxial Mesoderm

#### 2.1.1. Development of the Paraxial Mesoderm in Embryo

During gastrulation, the mesoderm is formed in the primitive streak and tail bud [16]. The mesoderm is divided into three populations: the paraxial mesoderm, intermediate mesoderm, and lateral plate mesoderm [17]. The paraxial mesoderm forms somites through somitogenesis regulated by a periodic segmentation clock, which subsequently develops into the sclerotome and dermomyotome [18]. Various tissues related to the axial skeleton originate from the sclerotome: the vertebrae, tendons, portions of the dorsal aorta and intervertebral blood vessels, and even the meninges [18,19]. The dermomyotome gives rise to the musculature and dermis [20].

In early mesodermal development and specification, the coordination of Wnt and bone morphogenic protein (BMP) signaling plays a key role. Wnt signaling promotes primitive streak formation and presomitic mesoderm differentiation [21,22]. The gradient of BMP activity generated by a BMP inhibitor, Noggin, expressed in the notochord and somitic mesoderm produces a mediolateral axis, which specifies the mesodermal fate. BMP signaling inhibits paraxial mesoderm formation but promotes intermediate and lateral plate mesoderm formation. This finding was demonstrated with chick embryos: The higher the BMP activity, the greater the induction of the lateral plate mesoderm [23].

The presomitic mesoderm forms somites through somitogenesis, which is orchestrated by the segmentation clock, determination front, and mesenchymal-epithelial transition [24]. The process of somitogenesis is restrictively and periodically regulated by several signaling pathways, including Notch, fibroblast growth factor (FGF), and Wnt [25]. Subsequently, the somite further develops into two derivatives, the sclerotome and the dermomyotome. The ventromedial part of the somite forms the sclerotome via epithelial-mesenchymal transition, whereas the epithelial dorsolateral part of the somite forms the dermomyotome. This specification process is dependent on the activity of morphogens such as sonic hedgehog (SHH), BMP, and Wnt [26].

#### 2.1.2. Recapitulating Development of the Paraxial Mesoderm in a Dish

**Figure 2:**
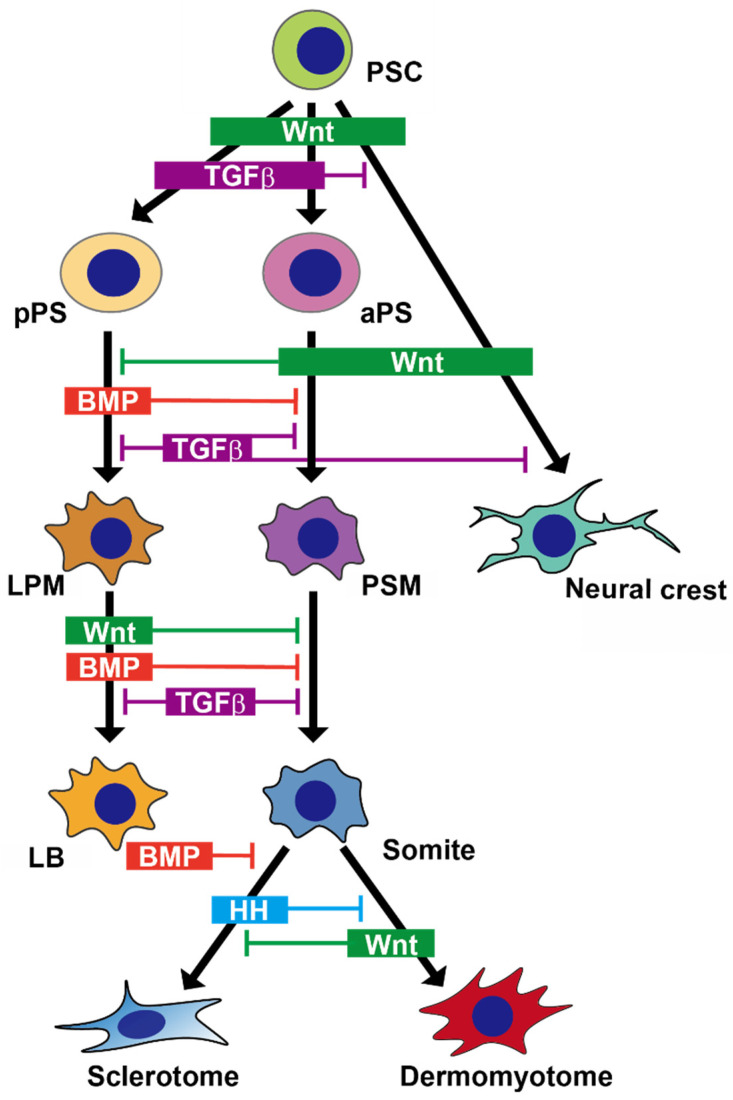
Schema of the induction process from human PSC to each derivative. Using a combination of major morphogens, mesoderm or neural crest derivatives can be induced in vitro. TGFβ; Transforming growth factor beta; BMP, bone morphogenic protein; HH, hedgehog; PSC, pluripotent stem cell; aPS, anterior primitive streak; pPS, posterior primitive streak; PSM, presomitic mesoderm; LPM, lateral plate mesoderm; LB, limb bud.

Several groups have already established stepwise protocols to induce either sclerotome or dermomyotome from hPSCs (Figure 2) [14,15]. In the primitive streak and paraxial mesoderm development, Wnt signaling plays a crucial role in inducing these cell types. Thus, Wnt activators such as CHIR99021, a GSK3 inhibitor, are often used in the first step to induce the primitive streak from both mouse PSCs (mPSCs) and hPSCs in vitro [5,14,22,27,28]. The additional FGF signaling with Wnt signaling enhances primitive streak induction, which has been demonstrated in various reports [5,14,15,22,29]. Transforming growth factor beta (TGFβ) signaling is another important pathway in early paraxial mesoderm development. However, TGFβ signaling in the presomitic mesoderm formation likely plays different roles in humans and other animals. TGFβ signaling was downregulated during the presomitic mesoderm differentiation process in humans but not in animals [22]. A TGFβ activator, Nodal, promoted the anterior primitive streak induction [5,14,30], whereas the inhibition of TGFβ with the activation of Wnt signaling enhanced the induction of human presomitic mesoderm [15,22]. In addition to Wnt activators, a BMP inhibitor, such as LDN-193189, also enhanced paraxial mesoderm differentiation with the upregulation of specific markers, including *TBX6* and *MSGN1* [5,14,15,22,29,31]. This efficacy of BMP inhibitor is possibly explained by the role of BMP signaling in mesoderm development. BMP activity inhibits paraxial mesoderm differentiation and promotes the intermediate or lateral plate mesoderm. The inhibition of BMP signaling also enhanced somite specification into the sclerotome [15,28]. Conversely, exogenous BMP signaling promoted the lateral plate mesoderm fate but suppressed the paraxial mesoderm fate [14]. In line with these findings regarding the roles of TGFβ and BMP signaling, some reports have shown that the inhibition of both TGFβ and BMP signaling enhances the induction efficacies of the presomitic mesoderm and somite formation from hPSCs [5,14,15,22,29].

#### 2.1.3. Chondrocyte Differentiation through the Paraxial Mesoderm in a Dish

During the developmental process, a large part of the sclerotome differentiates into chondrocytes. Most axial skeletons derived from the sclerotome are formed through endochondral ossification [24]. Thus, chondrocyte induction via sclerotome in vitro may be a straightforward process. Various reports have shown the protocol of chondrogenesis with the sclerotome, indicating that the sclerotome is a promising cell source for cartilage [5,14,15]. Although some other protocols did not dissect the details of the developmental stages [32,33,34], these protocols may recapitulate similar mesoderm development. Given the high induction rates of chondrocytes in these methods, there are multiple pathways to generate chondrocyte differentiation, at least under in vitro conditions. In addition, the combination of chondrogenic inducers is another key factor required for efficient chondrocyte induction. These include BMPs (BMP2, 4, and 7), TGFβ (b1, 2, 3, and Activin-A), GDF5, and others [35,36,37,38].

Although chondrocytes were reported to be induced under both 2D and 3D culture conditions, 3D culture seems to be more potent [39]. A study showed that hypertrophic chondrocyte marker genes were upregulated under 2D culture conditions in human articular chondrocytes, whereas chondrogenic markers were downregulated under the same conditions [40]. This result indicates that 3D culture methods yield less hypertrophic phenotypes than those derived in monolayers.

#### 2.1.4. Osteoblast Differentiation through the Paraxial Mesoderm in a Dish

Several protocols have been proposed for the stepwise induction of osteoblasts from the mesoderm or sclerotome derived from hPSCs and mPSCs [6,15,22,41,42]. In many cases, the conventional osteogenic medium is used as the basal medium containing fetal bovine serum, ascorbic acid, β-glycerophosphate, and dexamethasone [42,43]. Osteogenic inducers are used with the basal medium. These include BMPs, vitamin D3, TGFβ s [44], FGFs, hedgehog agonists, and Wnt agonists [42]. Manufactured products, whose components are not completely open to the public, are also often used for osteogenic induction [15,22]. Some studies have attempted to recapitulate the osteoblast differentiation in a stepwise manner [22,41,42], whereas a few studies have proposed direct conversion or induction into osteoblasts from either human somatic cells or even hPSCs [43,45,46,47].

Several studies have conducted 3D culture for osteoblast differentiation. We and others showed that 3D culture with scaffolds enhanced osteoblast differentiation in both mouse and hPSC-derived mesodermal cells [42,48,49]. Although no methods currently recapitulate the induction of vascularization into the induced bone structure in vitro, some reports have achieved such induction by utilizing an in vivo environment in mice. By implanting hPSC-derived sclerotome or cartilaginous particles into subcutaneous space or renal capsules of immunodeficient mice [5,14,33], the cells underwent endochondral ossification-like processes, generating columnar structures of chondrocytes and bone collar, and bone marrow-like structures [5,33].

Overall, the induction protocols for paraxial mesoderm derivatives have been well established, providing promising cell sources for bone and cartilage regeneration. The next challenge for the application of cell therapy will be the purification of progenitors or mature cells. In consideration of the risk of oncogenicity, completely removing undifferentiated cells is essential. Pharmacological purifications or other methods will be required for the clinical settings.

### 2.2. Lateral Plate Mesoderm

#### 2.2.1. Development of the Lateral Plate Mesoderm in Embryo

The lateral plate mesoderm gives rise to appendicular skeletons (except for musculature, which is derived from the dermomyotome via the somatic mesoderm) and the circulatory system, including the heart, blood vessels, and blood cells, via the splanchnic mesoderm [20,50]. The lateral plate mesoderm is derived from the posterior primitive streak and has common progenitors with the paraxial mesoderm: the BMP gradient produced by Noggin can create the bifurcation of the lateral plate and paraxial mesoderm [23]. Early specification of the forelimbs and hindlimbs occurs in the limb fields prior to limb bud formation based on the antagonism between retinoic acid (RA) and Fgf8. Studies with mouse and chick embryos demonstrated that RA is indispensable for upregulation of *Tbx5*, which is necessary for the forelimb specification and formation [51,52]. In addition to RA signaling, Wnt, FGF, and BMP signaling work cooperatively to regulate limb bud formation. Wnt signaling (Wnt3a in chickens, Wnt3 in mice and humans) induces Fgf8, which is necessary for apical ectodermal ridge (AER) formation. BMP signaling sufficiently regulates *En1* expression, which is also necessary for AER induction, in the ventral ectoderm [53,54,55]. Shh from the posterior mesenchyme (zone of polarizing activity, ZPA) modulates Fgf4 from posterior AER via the BMP antagonist, Gremlin1, which is important for *Fgf4* and *Fgf8* expression in the AER. Fgf4 maintains the polarizing region (Shh/Fgf4 feedback loop), which promotes growth and patterning of AER [56]. In turn, Fgf8 from AER triggers the Fgf/Grem1 inhibitory loop, which represses Gremlin1 [57]. This inhibitory loop terminates the outgrowth of AER, which may lead to attainment of proper tissue size [57]. Through limb formation, BMP promotes cartilage formation. BMP is also involved in bone development with Wnt/β-catenin signaling and apoptosis with FGF signaling from AER, respectively [58].

#### 2.2.2. Recapitulating Development of the Lateral Plate Mesoderm in a Dish

Based on the importance of BMP signaling in the limb bud development in vivo, BMP recombinant proteins have been used both to promote induction of the lateral plate mesoderm and to inhibit paraxial mesodermal differentiation in vitro [14,59]. Although Wnt activation promotes paraxial mesodermal differentiation, several studies used Wnt activation with a GSK3 inhibitor, CHIR 99021, in order to induce the primitive streak effectively [14,60]. Because TGFβ specifies the endoderm in the primitive streak, a TGFβ inhibitor was also used in some studies to block endoderm formation and to induce mesoderm instead (Figure 2) [14,60].

The protocols for limb bud induction vary. One study showed that Wnt signaling activation promoted limb bud specification and inhibited cardiac mesoderm differentiation [14]. Another study with mouse ESCs (mESCs) showed that 3D spheroid culture induced limb buds only with BMP4 [59]. The same study also demonstrated forelimb/hindlimb specification with RA and induction of AER-like tissues with the sequential combination of BMP inhibitor and Wnt agonist [59]. In addition, implantation of induced limb bud tissue into renal capsules of immunodeficient mice formed ectopic bone tissues recapitulating endochondral ossification [59].

#### 2.2.3. Chondrocyte and Osteoblast Differentiation through the Lateral Plate Mesoderm in a Dish

The lateral plate mesoderm, or its derivative limb buds, differentiate into various skeletal components, including chondrocytes and osteoblasts. Several reports have shown chondrocyte induction via the lateral plate mesoderm induced from mESCs in vitro or in vivo. Chondrocytes were induced from the mESC-derived lateral plate mesoderm in vitro under a condition of high cellular density with TGFβ3 and BMP2 supplementations [61]. Another report showed that organoids of limb buds derived from mESCs formed premature cartilage in vitro under a 3D culture condition [59]. In addition, the same study showed that the induced organoids were implanted into renal capsules of immunodeficient mice and formed bone and cartilage tissues [59]. The number of reports focusing on osteoblast induction via the lateral plate mesoderm is also limited. One study showed osteoblast induction via induced lateral plate mesoderm from human iPSCs (hiPSCs) by using manufactured osteogenic medium [6]. Overall, the protocols for the induction of skeletal cells from lateral plate mesoderm are still limited. Because limb bud-derived skeletal tissues are often related to skeletal diseases, including osteoarthritis and bone fracture [1], generating the “on-site” cell sources derived from lateral plate mesoderm may be promising.

### 2.3. Neural Crest

#### 2.3.1. Development of the Neural Crest in Embryo

The neural crest is derived from the ectoderm and gives rise to various tissues, including a large part of the facial skeleton, peripheral nervous system, adrenomedullary cells, and pigment cells [62]. Neural crest formation is initiated during the period of gastrulation. Presumptive neural crest territory forms at the neural plate border between the future neural and non-neural ectoderm. This border also gives rise to the placodes that generate tissues related to the eyes and ears in the anterior region. This border is specified by the orchestration of several signals, such as Wnts, BMPs, and FGFs produced by the ectoderm and mesoderm [63]. Previous studies have demonstrated that neural crest specification is sequentially regulated by signaling pathways and key transcription factors. First, Wnt and BMP signaling regulate the expression of genes that specify the neural plate border, including *Zic1*, *Msx1*, and *Tfap2* [64]. Second, the expression of genes that specify the neural crest fate is induced, including *FoxD3*, *Snai1/2*, and *Pax3/7* [63]. These neural crest specifiers activate the program of the epithelial-to-mesenchymal transition, which enables neural crest cells (NCCs) to develop into a migratory cell type via delamination from the ectoderm. In addition, these transcription factors are thought to maintain the properties of NCCs by coordinating with one another [63,65].

The neural crest is divided into four anatomical regions with unique characteristic derivatives and properties. (1) Cranial or cephalic NCCs give rise to the craniofacial skeleton and other tissues of the head and neck, including dental papilla in the tooth germ, cranial neurons, glia, pigment cells, and connective tissues [66]. (2) Cardiac NCCs contribute to the formation of outflow tracts of large arteries and aortopulmonary septation [67]. (3) Trunk NCCs migrate ventrolaterally and give rise to sensory neurons of the dorsal ganglia. They also migrate more ventrally and give rise to the sympathetic ganglia and the adrenal medulla [68]. However, trunk NCCs migrating dorsolaterally through the dermis develop into melanocytes [68]. (4) Vagal and lumbosacral NCCs give rise to the parasympathetic ganglia of the gut [66]. NCCs have the plasticity to differentiate into proper cell types according to the environment, and there are specific mechanisms regulating their multipotency. For example, cranial NCCs are distinct from trunk NCCs in terms of their differentiation potentials. Cranial NCCs can give rise to bone, cartilage, and muscles, whereas trunk NCCs cannot. *Hox* genes play a key role in this regulation, resulting in such regional differences. For example, *Hox* genes need to be fully repressed for skull formation in mice and chicks [69,70].

#### 2.3.2. Recapitulating Development of the Neural Crest and Its Derivatives in a Dish 

Several reports showed that NCCs were induced effectively from hPSCs by a combination of Wnt activation and TGFβ inhibition, although Wnt activation alone was also reported to induce the NCCs (Figure 2) [71,72]. The induced NCCs were maintained for more than 10 passages by using either of two specific combinations of reagents: the combination of a TGFβ inhibitor, EGF, and FGF2, or the combination of a GSK3 inhibitor and a TGFβ inhibitor [71,73]. Notably, this induction protocol is partially overlapped with a protocol to induce mesoderm. A recent study suggested that, in the trunk territory of the embryo, the neural crest may arise from an axial progenitor or neuromesodermal precursors [74]. This may be the reason why the early steps of induction protocols for mesoderm and neural crest are similar.

Recently, some research groups proposed that hiPSC-derived NCCs were applicable for chondrocyte induction via MSC-like cells [37,75]. In these reports, MSC-like cells were induced via NCCs using a relatively simple medium: aMEM supplemented with 10% fetal bovine serum (FBS) and 5ng/mL bFGF, or DMEM/F12 supplemented with 10% FBS. Similarly, osteoblast inductions were also performed via MSC-like cells derived from hiPSC-derived NCCs with manufactured osteogenic medium [37,75].

Because of the multipotency of NCCs, giving rise to various cell types in vivo, induced NCCs in vitro are promising cell sources for derivative-related disorders, such as involving peripheral nerves, corneas, teeth, and melanogenesis [76]. In addition, several research groups have proposed that NCC-derived cells/tissues can be applied to repair not only NCC-derived craniofacial skeletons, but also mesoderm-derived skeletal tissues [77]. Further characterization and validation of the induced NCCs and their derivatives will be necessary for these applications.

## 3. MSCs

In addition to the PSC-derived cells, other cell sources can be utilized for skeletal regeneration. These cells are mainly isolated from bone tissues. MSCs are the most conventional cell type and have been widely studied. MSCs were first harvested from the bone marrow [78]. Other than the bone marrow, various human tissues have been reported as sources of MSCs: adipose tissue, dermis, skeletal muscles, synovial membranes, saphenous veins, dental pulps, periodontal ligaments, Wharton’s jelly, umbilical cords including blood, amniotic fluid, lung, and liver [79]. Thus, MSCs contain a broad spectrum of cell types that have different potentials or functions. Because of the variety of cell sources, a minimal criterion for defining MSCs was proposed by the International Society for Cellular Therapy: (1) MSCs must be plastic-adherent when maintained under standard culture conditions; (2) MSCs must express CD105, CD73, and CD90 and lack expression of CD45, CD34, CD14 or CD11b, CD79a or CD19, and HLA-DR surface molecules; and (3) MSCs must differentiate into osteoblasts, adipocytes, and chondroblasts in vitro [80]. The international society for Cellular Therapy also proposed to call these cells multipotent mesenchymal stromal cells rather than mesenchymal stem cells [80].

MSCs have various biological functions. First, MSCs have multipotency to differentiate into a variety of cell types, including not only skeletal tissues but also other types of tissues, such as muscles and neurons [81,82]. Second, MSCs secrete cytokines that are thought to have a positive impact on the treatment of various diseases [83,84]. Third, MSCs suppress immune responses via apoptosis by recipient cytotoxic cells, which is an essential process for MSC-induced immunosuppression [85,86]. Recent studies suggested that the majority of the therapeutic potential arises from their paracrine and immunomodulation activities rather than their differentiation potencies [83,84]. Other reports have shown that MSCs have a hypo-immunogenic character by which they avoid recognition by immune cells and reduce their alloreactivity [87,88]. Thus, MSCs can serve as not just a cell source but also a producer of various reactive reagents. Indeed, MSCs have already been applied to clinical trials for various diseases: bone and cartilage diseases [89,90,91], cardiovascular diseases, neurological diseases [79], and graft-versus-host disease (GvHD) [10,92].

Despite the various potentials of MSCs, there are still some issues that must be addressed before applying them in clinical settings. First, harvesting MSCs usually requires invasive procedures. Second, the number of MSCs that can be sourced from a single donor will necessarily be limited. Third, the biological properties of MSCs for proliferation and differentiation will vary among donors and will be qualitatively and quantitatively limited in most cases [93]. To overcome these limitations, recent studies have attempted to generate MSCs from hPSCs. Several protocols have been proposed for the induction of MSCs from hPSCs [10,94,95]. Further analysis to test the integrity of the induced cells, including cell characterization and testing of their biological functions in vivo, will be important for the clinical usage.

## 4. SSCs

In addition to MSCs, another type of stem cells known as SSCs may be a candidate for skeletal regeneration. Although SSCs have been investigated, they have not been applied in clinical settings yet. Several recent studies have proposed that SSCs with different definitions in different contexts. SSCs can be divided into two classes: those which contribute to skeletal development, and those which contribute to skeletal homeostasis and regeneration in adults [11,96,97]. Here, we first focus on the SSCs proposed by Chan et al. [11,98], because these cells have been well defined with a combination of cell-surface markers. Chan et al. isolated mouse SSCs (mSSCs) and human SSCs (hSSCs) from the growth plate using different combinations of cell-surface markers: CD45^−^Ter119^−^Tie2^−^Thy1^−^6C3^−^CD51^+^CD105^−^CD200^+^ for mSSCs, and CD235^-^CD45^-^Tie2^-^CD31^-^PDPN^+^CD146^-^CD73^+^CD164^+^ for hSSCs. Both mSSCs and hSSCs have a self-renewal property that is evidenced by the combination of in vivo implantation with labeling and in vitro colony formation experiments. These cells have multipotency to differentiate into osteoblasts, chondrocytes, and stromal cells, but not adipocytes, as evidenced by cell culture analysis in vitro and clonal analysis with transplantation into immunodeficient mice. Chan et al. further identified BCSP (bone, cartilage, and stromal progenitors) with specific cell-surface makers. BCSP is an intermediate differentiation stage between SSCs and the committed cell types [11,98].

Notably, residential hSSCs as well as mSSCs demonstrated an injury-induced expansion in clinical specimens and xenograft experiments with human fetus phalanx, which may represent a regenerative response to skeletal injuries [11,99,100]. In addition, another report showed that co-delivery of BMP2 and soluble VEGFR1 promoted articular cartilage regeneration by activating endogenous mSSCs in knee joints [100]. These results suggest that manipulation of the endogenous SSCs properties by reagents including growth factors and small compounds may be a therapeutic option for skeletal injuries and possibly degenerative diseases, such as osteoarthritis and osteoporosis. In addition to the isolation of cells from adult tissues or manipulation of the endogenous cells, the research group proposed an in vitro differentiation protocol from hPSCs to hSSCs, suggesting these cell sources can be generated and expanded in vitro [11].

There are other SSCs that were identified in different ways by other research groups mainly by lineage tracing analysis in mice. These include SSCs located in the bone marrow and defined as *Nestin*^+^ [101], *Grem1*^+^ [102], *Cxcl12*^+^ [97], or *LepR*^+^ [96]; SSCs located in the growth plate and defined as *PTHrP*^+^ [103,104]; SSCs located in the perichondrium and defined as *Axin2*^+^ [105]; and SSCs located in the periosteum/endosteum and defined as *Ctsk*^+^ [106]. Although these cells are promising cell sources for regenerative medicine, further analysis in human studies will be required. Identifying the corresponding cell types in human tissues, characterization of the cells, ideally by using cell-surface markers, and testing of the multipotency and efficacy for skeletal regeneration will be important for the cell therapy.

## 5. CAR Cells

CAR cells are another putative cell source for skeletal homeostasis and regeneration. CAR cells are located in adult bone marrow and have multipotency to differentiate into osteoblasts and adipocytes, but not chondrocytes. Thus, one might imagine that CAR cells are overlapped with or a subpopulation of SSCs or bone marrow MSCs [107]. However, *Cxcl12*-expressing cells include not only an SSC-like population but also osteoblasts and endothelial cells in mice [108]. For this reason, it would probably be better not to classify CAR cells as SSCs.

A key property of CAR cells is the maintenance of hematopoietic stem cells (HSCs) as a niche [109]. Ablation of CAR cells using CXCL12-DTR-GFP mice with diphtheria toxin showed not only impaired adipogenic and osteogenic differentiation but also decreased HSCs, lymphoid and erythroid progenitors [12]. Moreover, another recent study showed that *Cxcl12*-positive bone marrow cells and their descendants largely contributed to cortical bone regeneration in a femur injury model in mice [97]. Notably, this bone regeneration was dependent on the activation of canonical Wnt signaling, whereas it was independent of expressions of *Sox9* or *Runx2*, master transcription factors in bone development [97]. This result suggests that CAR cell-mediated bone regeneration may be distinct from bone regeneration resembling embryonic skeletal development. Currently, there is no published protocol to induce, expand, and maintain CAR cells in vitro. Clinical application of human CAR cells for cell therapy has not been done yet. Given the wide-range of clinical potentials of CAR cells, including hematopoiesis and osteogenesis, such investigations will be needed in the near future.

## 6. Summary and Future Perspectives

Overall, there are various cell sources having unique properties and attractive potentials for skeletal regeneration. Some are generated from pluripotent stem cells; others are isolated from skeletal tissues. Several protocols have been established for inducing skeletal cells from pluripotent stem cells through distinct origins of bone. One of these, a protocol for inducing allogenic iPSC-derived cartilage graft to heal articular cartilage injury, has already been advanced to clinical trial (https://jrct.niph.go.jp/en-latest-detail/jRCTa050190104) (Table 1). In the context of stem cells from skeletal tissues, MSCs have been widely used in the research field for a long time, and clinical trials have been conducted or are still ongoing (Table 1). Other stem cells from skeletal tissues have not yet been assessed for regenerative properties in clinical trials.

In the future, the properties of these stem cells need to be further elucidated for application. Different types of injury or defects may need distinct cellular properties of stem cells for the regeneration. One may predict that high proliferation and differentiation capacity of multiple skeletal cell types are required for bone regeneration, whereas the regeneration of articular cartilage may need different cellular properties. Not cells, but cytokines from stem cells, or the cell populations arising from stem cells may be required for specific injury/regeneration. Thus, further understanding of the molecular mechanisms underlying tissue regeneration will facilitate not only better choice of stem cell types but also promote further optimization of cell therapy. Assessment of the skeletal regeneration among different cell sources provides clues to establish the type of injury or defect requiring distinct cell therapy strategy.

The next step of regenerative strategy with pluripotent stem cells will be a comparative analysis of the induced cells in terms of their regenerative potential for cell therapy. One might expect that the regenerative capacity would be best suited for bone defect sites in an origin-dependent manner. For example, neural crest-derived osteoblasts may be suitable for bone defects in calvaria; whereas lateral plate mesoderm-derived skeletal cells may work well for femur fractures. A recent study compared these three origin-derived osteoblasts and showed their origin-specific signatures [6]. Another consideration is that inducing rare but crucial skeletal cell types will be important. For example, cells in the superficial layers are reported to be progenitors in adult mouse articular cartilage; these cells are known to be degraded in osteoarthritis [110,111]. Thus, the establishment of an induction protocol for these clinically relevant cell types will be a next target.

There are several stem cells in bone marrow, and all of them would be promising cell sources for bone regeneration. However, these cells have been defined by different markers by different research groups; it is not clear whether a particular defined cell type is completely different from the others or whether it partially overlaps them. Thus, comparative analysis among these cell types will be required. One stem cell population may have a preference to differentiate into distinct cell types. Another population may have a high level of plasticity depending on the circumstances and conditions of the local environment where it is implanted. To test these possibilities, emerging technologies will be helpful. Single-cell analysis may address the heterogeneity of the cells expressing current maker genes. Molecular recording, which enables the tracing of multiple cell lineages by means of molecular barcodes and the CRISPR-Cas9 system, may address the trajectory of the cell lineages from stem cell states. We hope that further research will help us to characterize stem cells more precisely, optimize the preparation conditions, and identify a cell source most suited to the treatment of skeletal diseases.

## Figures and Tables

**Table 1:** Representative clinical trials for skeletal regeneration using stem cells. Ongoing studies were mainly picked up from UMIN Clinical Trials Registry (https://www.umin.ac.jp/ctr/index.htm) or Clinicaltrials.gov (https://clinicaltrials.gov/ct2/home) as of December 2020. Other trials can be found in the web sites. Each web link of which was listed here as follows.

Cell Type	Condition	Method	Cell Source	Phase
iPS ^(1)^	Knee articular cartilage damage	Implantation of iPSC-derived Cartilage	Alogenic (iPSCs)	N/A
MSC ^(2)^	Knee articular cartilage damage	Arthroscopy, Microfracture	Autologus (synovium)	N/A
MSC ^(3)^	Knee osteoarthritis	Intra-articular injection	Autologus (bone marrow)	1
MSC ^(4)^	Knee osteoarthritis	Transplantation with high tibial osteotomy	Alogenic (umbilical cord blood)	2
MSC ^(5)^	Knee osteoarthritis	Intra-articular injection	Autologus (adipose, bone marrow)	3
MSC ^(6)^	Knee osteoarthritis	Intra-articular injection	Autologus (adipose)	4
MSC ^(7)^	Osteoporotic Spinal fracture	Intravenous Infusion	Autologus (bone marrow)	1
MSC ^(8)^	Nonunion of Fracture	Injection at the fracture site	Autologus (adipose)	1, 2
MSC ^(9)^	Nonunion of Fracture	Implantation with biomaterial	Autologus (bone marrow)	3

^(1)^https://jrct.niph.go.jp/en-latest-detail/jRCTa050190104. ^(2)^https://clinicaltrials.gov/ct2/show/NCT02696876. ^(3)^https://clinicaltrials.gov/ct2/show/NCT03477942. ^(4)^https://upload.umin.ac.jp/cgi-open-bin/ctr_e/ctr_view.cgi?recptno=R000046638. ^(5)^https://clinicaltrials.gov/ct2/show/NCT04351932. ^(6)^https://clinicaltrials.gov/ct2/show/NCT04675359. ^(7)^https://clinicaltrials.gov/ct2/show/NCT02566655. ^(8)^https://clinicaltrials.gov/ct2/show/NCT04340284. ^(9)^https://clinicaltrials.gov/ct2/show/NCT03325504.
